# From nerves to brain to gastrointestinal tract: A time-based study of parrot bornavirus 2 (PaBV-2) pathogenesis in cockatiels (*Nymphicus hollandicus*)

**DOI:** 10.1371/journal.pone.0187797

**Published:** 2017-11-09

**Authors:** Jeann Leal de Araujo, Raquel R. Rech, J. Jill Heatley, Jianhua Guo, Paula R. Giaretta, Ian Tizard, Aline Rodrigues-Hoffmann

**Affiliations:** 1 Department of Veterinary Pathobiology, Texas A&M University, College Station, Texas, United States of America; 2 Department of Small Animal Clinical Sciences, Texas A&M University, College Station, Texas, United States of America; Division of Clinical Research, UNITED STATES

## Abstract

Parrot bornaviruses (PaBVs) are the causative agents of proventricular dilatation disease, however key aspects of its pathogenesis, such as route of infection, viral spread and distribution, and target cells remain unclear. Our study aimed to track the viral spread and lesion development at 5, 10, 20, 25, 35, 40, 60, 80, 95 and 114 dpi using histopathology, immunohistochemistry, and RT-PCR. After intramuscular inoculation of parrot bornavirus 2 (PaBV-2) in the pectoral muscle of cockatiels, this virus was first detected in macrophages and lymphocytes in the inoculation site and adjacent nerves, then reached the brachial plexus, centripetally spread to the thoracic segment of the spinal cord, and subsequently invaded the other spinal segments and brain. After reaching the central nervous system (CNS), PaBV-2 centrifugally spread out the CNS to the ganglia in the gastrointestinal (GI) system, adrenal gland, heart, and kidneys. At late points of infection, PaBV-2 was not only detected in nerves and ganglia but widespread in the smooth muscle and/or scattered epithelial cells of tissues such as crop, intestines, proventriculus, kidneys, skin, and vessels. Despite the hallmark lesion of PaBVs infection being the dilation of the proventriculus, our results demonstrate PaBV-2 first targets the CNS, before migrating to peripheral tissues such as the GI system.

## Introduction

Initially described in the late 1970s as a disease of large psittacine birds, and therefore originally named “macaw wasting syndrome”, proventricular dilatation disease (PDD) is a fatal and important disease of psittacine birds worldwide, however, its cause remained obscure for many decades [1, 2].

In 2008, two independent studies identified a group of enveloped, non-segmented, negative sense single-stranded RNA viruses of the *Bornaviridae* family as the cause of PDD [3, 4]. This virus was initially named avian bornavirus, but further molecular investigation revealed a diverse group of viruses [5–10]. The discovery of this high genetic variability of avian bornaviruses associated with the identification of the Variegated squirel bornavirus 1 (VSBV-1), caused an important rearrangement in the monogeneric family *Bornaviridae*, which was reorganized in 2015 to incorporate 8 distinct species of mammalian, reptile and avian, besides unclassified and unassigned bornaviruses [11]. Eight viruses have been identified in psittacine birds, named parrot bornavirus 1 to 8 (PaBV-1 to PaBV-8). Five of these viruses (PaBV-1 to 4, PaBV-7) belong to the species *Psittaciform 1 bornavirus*, whereas PaBV-5 is a member of the species *Psittaciform 2 bornavirus*. PaBV-6 and PaBV-8 remain unclassified [12].

Avian bornaviruses have been identified worldwide, including countries such as the United States, Canada, Brazil, South Africa, Austria, Germany, Spain, United Kingdom, Italy, Denmark, Australia, and Japan [13]. Psittaciform 1 and 2 bornaviruses affect different species of psittacine birds, such as amazon and grey parrots, macaws, cockatoos, cockatiels, and conures. While, passerine birds and waterfowl harbor their own avian bornaviruses, which are members of the species *Passeriform 1* and *2 bornaviruses* and *Waterbird 1 bornavirus*, respectively [2, 14]. These bornaviruses can affect a broad range of tissues and cell types, however, neurotropism is a pronounced feature, which is reflected by a lymphoplasmacytic meningoencephalomyelitis and ganglioneuritis (remarkably prominent in the enteric nervous system) [15–17], especially in psittacine birds. These lesions may give rise to neurologic or gastrointestinal signs, or even a combination of both, including lethargy, depression, ataxia, regurgitation, emaciation and impairment in digestion [4, 18–20], therefore, clinical presentation of PDD is markedly variable and recent studies suggest that different parrot bornaviruses (PaBVs) can cause different patterns of PDD lesions and clinical disease, with predominant neurological or predominant gastrointestinal presentations [20]. The development of the disease is also particularly variable, ranging from acute death to slow progression of clinical disease and not all infected psittacine birds develop clinical disease [20–22].

Despite the intensive efforts on PDD research, the pathogenesis of this condition and how PaBVs cause disease remain unclear. Several studies were able to reproduce clinical disease and classical lesions of PDD after experimental inoculation of PaBVs and recover this virus in birds that developed the disease, which fulfills Koch’s postulates and reaffirms PaBVs as the putative agent of PDD in psittacine birds [2, 8, 19, 20, 23, 24, 27, 28]. These studies were also able to study the course of avian bornaviruses by analysis of *intra vitam* samples such as swabs and serology. However, they did not evaluate the infection progress and sequence of tissues infected by the virus at different sequential timepoints. The aim of this study was to analyze the infection pathway after experimental inoculation of PaBV-2 in cockatiels using molecular, histological and immunohistochemical methods and to evaluate the viral presence and inflammation at early and later infection.

## Materials and methods

### Ethics statement

All procedures in this study were conducted using protocols approved by the Texas A&M Biosafety and Animal Use Committees (IACUC 20150–0045) and Institutional Biosafety Committee (IBC2015-021 and IBC2015-142), that meets all federal requirements, as defined in the Animal Welfare act (AWA), the Public Health Service Policy (PHS) and the Humane Care and Use of Laboratory Animals.

### Viral culture, titration and inoculum preparation

PaBV-2 isolate was inoculated into duck embryo fibroblasts (DEF, Schubot center laboratory cell collection) cultured for 7 passages in minimum essential medium (MEM) supplemented with 10% fetal bovine serum (FBS, Gibco, ThermoFisher Scientific, Walthan, MA), and subsequently maintained in MEM supplemented with 2% FBS until 70–80% cell confluence. The cells cultures were subjected to three cycles of freeze thaw and brief sonication. The cell debris were removed by centrifugation at 3000g for 10 minutes. Serial 10-fold dilutions of three stocks of virus aliquots were analyzed by focus-forming assays in order to determine viral titration. Titers above 8 x 10^5^ focus forming units per milliliter (FFU/ml) were considered acceptable for the experimental inoculation as previously described [23, 25].

### Experimental animals, virus inoculation, and infection timeline

**Fig 1:**
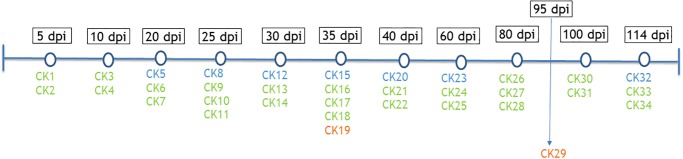
Distribution of infected (green) and control (blue) cockatiels throughout timepoints. Cockatiels that naturally died during the experiment are represented in orange.

Thirty-four cockatiels (*Nymphicus hollandicus*) originated from 2 breeders were used for experimental inoculation of PaBV-2 (CK1-34). Cloacal and choanal swabs from all cockatiels were tested weekly 3 times by RT-PCR in order to exclude the possibility of them being carriers of PaBVs. Twenty-seven cockatiels were inoculated with 0.1 ml of infected DEF lysates containing 8 x 10^4^ FFU of PaBV-2 in the right pectoral muscle. Seven cockatiels (CK5, CK8, CK12, CK15, CK20, CK23, and CK32) were inoculated only with 0.1 ml of PaBV-free DEF and served as negative controls. Cockatiels were divided into 12 different groups that corresponded to different euthanasia timepoints (5, 10, 20, 25, 30, 35, 40, 60, 80, 95, 100, and 114 days post-inoculation, dpi) (Fig 1). All cockatiels were evaluated twice a day for any alterations in the normal behavior and general health. Any clinical signs, in special the ones related to PDD (emaciation, anorexia, regurgitation, undigested seeds in the feces and neurological signs) were documented. At the correspondent timepoints or in case the animals presented neurological or/and gastrointestinal signs, cockatiels were humanely and painlessly euthanized with carbon dioxide (CO_2_) after being anesthetized with isoflurane (IsoThesia, Henry Schein, Melville, NY). Cockatiels were kept in cages with no more than 6 animals, with access to high-quality food, water, solar light and environmental enrichment. Animals with clinical signs of diseases other than PDD were excluded from the experiment and treated accordingly. One cockatiel originally obtained for this study had a self-inflicted traumatic injury and it was excluded from the experiment.

### Postmortem examination and sample collections

A complete necropsy was performed in each bird. During necropsies, samples from cloacal and choanal swabs, blood, site of inoculation (pectoral muscle), feather calamus, skin between the scapulae, skin from wing, skin behind the neck, crop, proventriculus, ventriculus, small intestine, large intestine, kidney, spleen, heart, liver, brain, spinal cord, brachial plexus and adrenal gland were collected for RT-PCR. Additionally, samples from skin behind the neck, skin between the scapulae, skin from the wing, skin from uropygium, site of inoculation (pectoral muscle), leg muscle, brachial plexus, vagus nerve, sciatic nerve, heart, thyroid and parathyroid glands, tongue, pharynx/larynx, thymus, esophagus, crop, proventriculus, ventriculus, small intestine, pancreas, large intestine, cloaca, spleen, liver, kidney, adrenal glands, trachea, syrinx, lungs, brain, spinal cord, eyes, head sinuses, tibiotarsus, and adjacent ganglia of all organs were also collected for histopathology and immunohistochemistry.

### Histopathology and immunohistochemistry

Samples collected during necropsy were fixed in 10% neutral buffered formalin and processed routinely for histopathology. A morphologic diagnosis indicating chronicity, severity, and nature of lesions was documented for each organ or tissue. Lymphoplasmacytic inflammation related to PaBV-2 infection was scored as follows: − (absent), + (minimal), ++ (mild), +++ (moderate), ++++ (severe). Immunohistochemistry was performed on the serial tissue sections, mounted on charged slides, and examined by light microscopy. Processing was done on an automated stain equipment, using rabbit polyclonal antiserum raised against PaBV-2 N protein. Briefly, after manual deparaffinization and rehydration, antigen retrieval was performed on a pressure cooker utilizing the commercial compound EMS buffer C, PH 4.5 (Electron microscopy sciences, Hatfield, PA) for 20 min under 121°C. Sections were treated with 0.3% hydrogen peroxide in order to block endogenous peroxidase activity for 45 minutes and with a protein blocker for endogenous protein activity for 10 minutes. Slides were incubated with rabbit anti-PaBV-N serum (1:1000) for 1 hour, followed by 10 minutes incubation with antibody enhancer and 15 minutes incubation with a goat anti-mouse/rabbit polymer visualization system (Thermo Fisher Scientific, Waltham, MA). Additionally, 3, 3'-Diaminobenzidine (DAB, Thermo Fisher Scientific) was used as a chromogen for color development. Slides were counter-stained with hematoxylin and examined under light microscopy. Polyclonal antibodies were developed by a commercial company (Lifetein, Somerset, NJ) against a specific region of the PaBV-2 N-protein. Due to the intranuclear replication nature of PaBVs, only cells with intranuclear or intranuclear and intracytoplasmic immunolabeling were considered positive. Cells only with intracytoplasmic immunolabeling were considered negative. Slides were scored based on the amount of positive cells in the section: − (absent), + (1–3% affected cells), ++ (4–10% affected cells), +++ (11–25% affected cells) and ++++ (more than 25% affected cells). The score of histopathology and immunohistochemistry for each timepoint was based on the average of the results of all birds in the respective timepoint. However, when only one bird presented inflammation or was positive by IHC, its score was considered representative for the timepoint. A complete list of results for each individual bird is available as supporting information (S1 Table).

### RNA extraction, RT-PCR and agarose gel electrophoresis

Collected samples were evaluated for presence of PaBV-2 RNA by RT-PCR detection of PaBV-2 matrix (PaBV-M) genes, as previously described [25]. Viral RNA was extracted from tissues using an RNeasy mini kit (Qiagen, Valencia, CA, USA), following manufacturer’s instructions. Viral RNA was reverse transcribed using high capacity cDNA synthesis kit (Applied Biosystems, Foster City, CA) and cDNA generated using random primers. Finally, PaBV-2 cDNA was amplified using the following primers targeting the M (Matrix) protein gene: PaBV M F (5-GGTAATTGTTCCTGGATGG-3) and PaBV M R (5-ACACCAATGTTCCGAAGACG-3). The PaBV-2-infected DEF used for viral culture were used as a positive control. After amplification, the PCR products were separated by size using electrophoresis in an agarose gel that was examined under ultraviolet light. Bands on the agarose gel were scored as positive (+) or negative (-). The timepoint was considered positive for PaBV-2 RNA by RT-PCR when at least one bird was positive in the given timepoint. Selected samples were also tested by qPCR as previously described elsewhere [25], and using primers and probes targeting the M protein gene: ABV M1F primer-5-GGTAATTGTTCCTGGATGG-3 (36 μM) and ABV M2R primer-5-GG[Y]TC[Y] [Y]TCACTGAAAGAAA[H]GG-3 (36 μM), and ABV M TaqMan Probe -5 FAM-CCAACAAAGTCTAT[Y]TCCA[R]C-3 -BHQ (10 μM), in order to compare sensitivity and confirm the conventional PCR results. Quantification cycles above 35 were considered negative, based on our standard curve threshold. A complete list of RT-PCR and qPCR results for each individual bird is available as supporting information (S2 Table).

## Results

### Clinical disease and macroscopic findings

**Fig 2:**
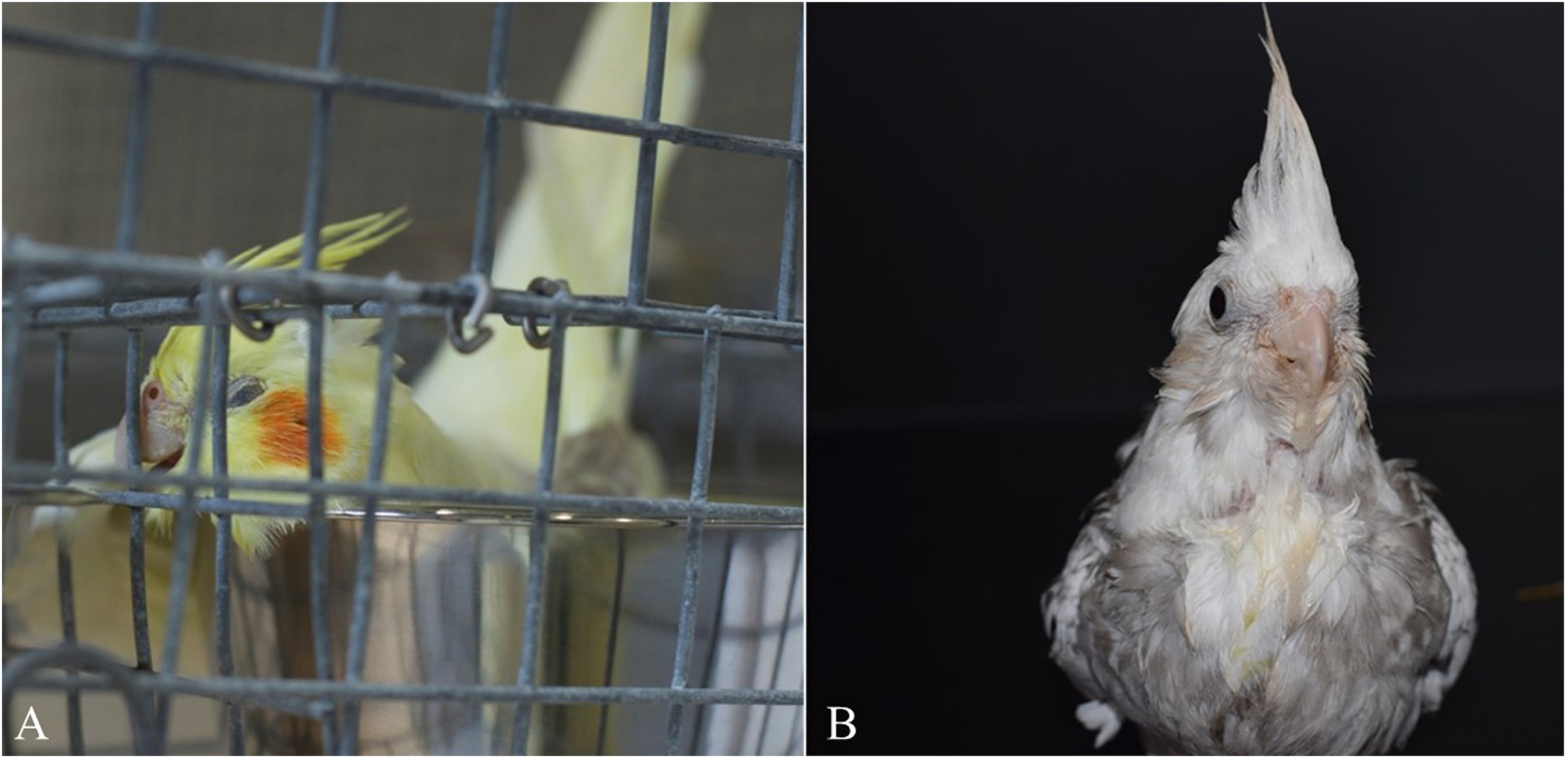
Clinical signs of cockatiels (*Nymphicus hollandicus*) infected with PaBV-2. Lethargy and depression of CK19 at 35 dpi (A) and ingesta-stained feathers in the foreneck and breast of CK24 (60 dpi) after regurgitation (B).

**Fig 3:**
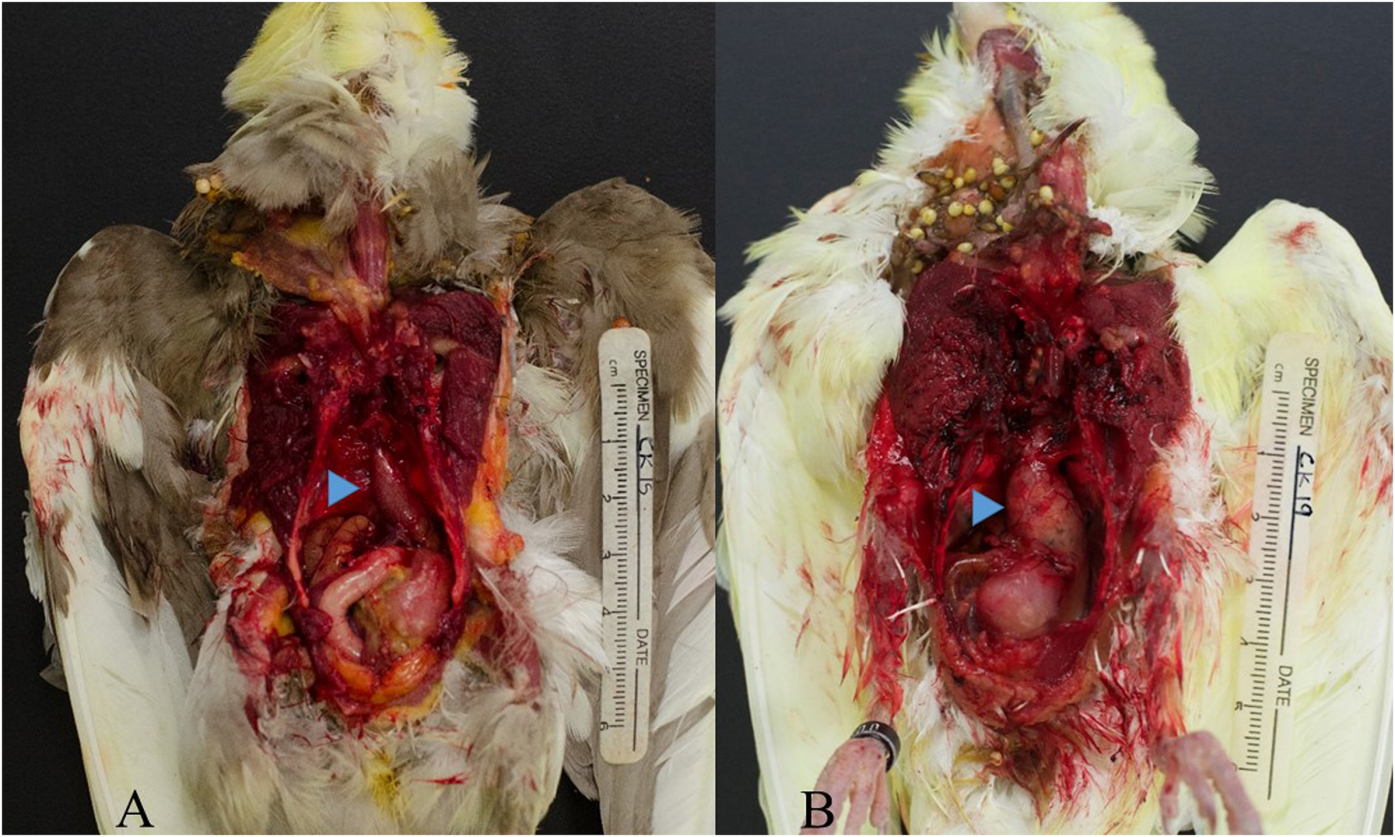
Gross findings. Comparison of proventriculus size (arrowheads) in a control (A) and infected (B) cockatiels of the same timepoint (35 dpi). Note the distention of the proventricular wall in CK19 when compared to a control cockatiel (CK15).

At 35 dpi, one of the cockatiels (CK19) presented with acute signs of depression, dyspnea, and lethargy (Fig 2A), and died shortly thereafter. A post-mortem examination was promptly performed. At 60 dpi, CK24 presented with mild signs of lethargy, ruffled feathers and regurgitation (Fig 2B) and euthanasia was elected, followed by necropsy. Additionally, CK29 was found dead at 95 dpi with no prior signs of illness. Macroscopic lesions were observed in 6 cockatiels (CK19, 23, 28, 29, 33, 34) and consisted of crop and/or proventricular dilatation (Fig 3B). None of the control birds presented clinical signs or macroscopic lesions.

### Microscopic findings

A summary of the microscopic lesions of PaBV-2 in infected cockatiels are shown in Table 1. Inflammatory lesions were observed as soon as 5 dpi in the site of inoculation, which was a consistent finding throughout all timepoints and most likely to be caused by the injection itself. This inflammatory infiltrate was characterized by multifocal aggregates of lymphocytes, plasma cells and macrophages and ranged from a minimal to severe myositis frequently organized around vessels, and extending to intercostal and pectoral nerves. The first lesions outside the site of inoculation were then observed in the brachial plexus at 20 dpi in CK6 and consisted of minimal lymphoplasmacytic neuritis. At 25 dpi, inflammatory lesions were observed in the cervical, thoracic and lumbar segments of the spinal cord and adjacent ganglia, as well as in the brain. Inflammation in the CNS was comprised of perivascular cuffing, mainly in the gray matter and meninges, with occasional gliosis and glial nodules at later timepoints. From 25 dpi and beyond, encephalitis and/or myelitis were consistently observed in all cockatiels. At 30 dpi, besides the CNS, inflammatory lesions were observed in the crop and intestinal ganglia, vagus nerve and in the adrenal medulla and adjacent ganglia. CK19, at 35 dpi, was not only the first cockatiel to develop clinical signs of PDD and die, but also the first cockatiel to present proventricular and ventricular ganglioneuritis and also lymphoplasmacytic infiltrates in the optic and sciatic nerves. These lesions were intermittently observed in the subsequent timepoints. At 40 dpi, the epicardial and renal ganglia, and nerves in the cloacal region presented the first inflammatory lesions. A lymphoplasmacytic dermal neuritis was observed at 80 dpi, and the skin from the wing was not only the first skin site to present inflammation but the most frequent one, being further observed at 95, 100, and 114 dpi. Dermal neuritis was observed in the skin from the uropygial gland (95, 100, and 114 dpi), skin behind the neck (80, 100, and 114 dpi) and skin between the scapulae (100 and 114 dpi). These histopathological findings were observed intermittently until 114 dpi and severity ranged from minimal to severe. No lesions related to PaBV-2 infection were observed in the liver, spleen, tongue, leg muscle, trachea, lungs, head sinuses, syrinx, thyroid and parathyroid glands, tibiotarsus, or thymus of any infected cockatiels. Additionally, no inflammatory lesions associated with bornavirus infection were observed in any tissues of the control cockatiels.

**Table 1:** Summary of inflammatory lesions, PCR and IHC results observed in 12 sequential timepoints following days post infection.

	5 dpi			10 dpi			20 dpi			25 dpi			30 dpi			35 dpi			40 dpi			60 dpi			80 dpi			95 dpi			100 dpi			114 dpi		
Tissue	INF	IHC	PCR	INF	IHC	PCR	INF	IHC	PCR	INF	IHC	PCR	INF	IHC	PCR	INF	IHC	PCR	INF	IHC	PCR	INF	IHC	PCR	INF	IHC	PCR	INF	IHC	PCR	INF	IHC	PCR	INF	IHC	PCR
Site of inoculation	+	+		+	+		+++	+	+	+++	+	+	+	+	+	+	+	+	++	+	+	+++	+	+	++	+	+	+	+	+	+	-	+	++	-	+
Brachial plexus	-	-	-	-	-	-	-	+	+	-	+	+	-	+	+	-	+	+	+	++	+	++	+	+	+	+	+	+	+	+	-	++	+	-	++	+
Spinal cord	-	-	-	-	-	-	-	+	+	++	+	+	++	++	+	+++	++	+	++	+++	+	+++	+++	+	+++	+++	+	++	+++	+	++	+++	+	+++	+++	+
Brain	-	-	-	-	-	-	-	-	-	++	+	+	+	+++	+	++	+++	+	++	+++	+	+++	+++	+	++	+++	+	+	+++	+	++	++++	+	++	++++	+
Sciatic nerve	-	-		-	-		-	-		-	-		-	-		++	-		-	+		+	+		+	+		+	+		+	+++	+	+	+++	+
Vagus nerve	-	-		-	-		-	-		-	-		-	-		++	-		-	++		+	++		-	++		-	++		-	+++	+	+	+++	+
Crop	-	-	-	-	-	-	-	-	-	-	-	-	-	+	+	++	+	+	++	++	+	++	++	+	+++	++	+	+	++	+	++	+++	+	+++	+++	+
Esophagus	-	-		-	-		-	-		-	-		-	-		-	-		-	+		++	+		-	+		-	+		-	+++	+	++	+++	+
Proventriculus	-	-	-	-	-	-	-	-	-	-	-	-	-	-	-	+++	-	-	+	+++	+	+++	+	+	++	+	+	-	+	+	+++	+++	+	++	+++	+
Ventriculus	-	-	-	-	-	-	-	-	-	-	-	-	-	-	-	++	-	-	++	++++	+	++++	+++	+	++	+++	+	+	+++	+	+++	++++	+	++	++++	+
Small intestine	-	-	-	-	-	-	-	-	-	-	-	-	-	+	+	++	+	+	++	++	+	+++	++	+	++	++	+	-	++	+	++	+++	+	+++	+++	+
Large intestine	-	-	-	-	-	-	-	-	-	-	-	-	-	+	+	+++	+	+	++	++	+	++	+++	+	++	+++	+	-	+++	+	++	++++	+	+	++++	+
Pancreas	-	-		-	-		-	-		-	-		-	-		-	-		-	-		-	+		-	-		-	-		-	+	+	-	++	+
Cloaca	-	-		-	-		-	-		-	-		-	-		-	-		++	++		++	+		-	-		++	+		++	++	+	++	++	+
Adrenal gland	-	-	-	-	-	-	-	-	-	-	-	-	-	+	+	++	+	+	++++	++	+	++	++	+	++	+++	+	+++	+++	+	+++	++++	+	+++	++++	+
Kidney	-	-	-	-	-	-	-	-	-	-	-	-	-	-	-	-	-	-	+++	++	+	++	+	+	++	+	+	++	+	+	++	+	+	+++	++	+
Heart/Great vessels of the heart	-	-	-	-	-	-	-	-	-	-	-	-	-	-	-	-	-	-	++	+++	+	++	+++	+	++	++	+	++	++	+	++	+++	+	+++	+++	+
Feathered skin (Wing)	-	-	-	-	-	-	-	-	-	-	-	-	-	-	-	-	-	-	-	-	-	-	-	+	++	++	+	+	+	+	++	+++	+	++	+++	+
Feathered skin (Behind the neck)	-	-	-	-	-	-	-	-	-	-	-	-	-	-	-	-	-	-	-	-	-	-	-	-	+	++	+	-	-	-	++	+++	+	++	++++	+
Feathered skin (Between scapulae)	-	-	-	-	-	-	-	-	-	-	-	-	-	-	-	-	-	-	-	-	-	-	-	-	-	-	+	-	-	-	++	++	+	++	++	+
Feathered skin with uropygial gland	-	-		-	-		-	-		-	-		-	-		-	-		-	-		-	-		-	-		+	+		++	++	+	++	++++	+
Feather calamus	-	-	-	-	-	-	-	-	-	-	-	-	-	-	-	-	-	-	-	-	-	-	-	-	-	-	-	-	-	-	-	-	-	++	++++	+
Eyes	-	-		-	-		-	-		-	-		-	-		+	+		+	+		+	+		++	+		+	+		+	+		+	+	
Cloacal swab			-			-			-			-			-			+			+			+			+			+			+			+
Choanal swab			-			-			-			-			-			-			-			+			+			+			+			+
Tongue	-	-		-	-		-	-		-	-		-	-		-	-		-	-		-	-		-	-		-	-		+	-		++	-	
Spleen	-	-	-	-	-	-	-	-	-	-	-	-	-	-	-	-	-	-	-	-	-	-	-	-	-	-	-	-	-	-	-	++	+	-	++	+
Leg muscle	-	-		-	-		-	-		-	-		-	-		-	-		-	-		-	-		-	-		-	-		-	-		++	-	
Liver	-	-	-	-	-	-	-	-	-	-	-	-	-	-	-	-	-	-	-	-	-	-	-	-	-	-	-	-	-	-	-	-	-	-	-	-
Pharynx/Larynx	-	-		-	-		-	-		-	-		-	-		-	-		-	-		-	-		-	-		-	-		-	-		-	-	
Thyroid/Parathyroid gland	-	-		-	-		-	-		-	-		-	-		-	-		-	-		-	-		-	-		-	-		-	-		-	-	
Thymus	-	-		-	-		-	-		-	-		-	-		-	-		-	-		-	-		-	-		-	-		-	-		-	-	
Trachea	-	-		-	-		-	-		-	-		-	-		-	-		-	-		-	-		-	-		-	-		-	-		-	-	
Syrinx	-	-		-	-		-	-		-	-		-	-		-	-		-	-		-	-		-	-		-	-		-	-		-	-	
Lungs	-	-		-	-		-	-		-	-		-	-		-	-		-	-		-	-		-	-		-	-		-	-		-	-	
Head sinuses	-	-		-	-		-	-		-	-		-	-		-	-		-	-		-	-		-	-		-	-		-	-		-	-	
Tibiotarsus+Bone marrow	-	-		-	-		-	-		-	-		-	-		-	-		-	-		-	-		-	-		-	-		-	-		-	-	
Blood			-			-			-			-			-			-			-			-			-			-			-			-

An average of the results for the cockatiels from each group was used to provide the timepoint information. Samples were considered positive when at least one of the samples in the respective timepoint was positive. Blanks indicate non-applicable sample or sample not evaluated for the mentioned technique. Individual results for each cockatiel are available as supporting information (S1 Table).

### Immunohistochemistry

**Fig 4:**
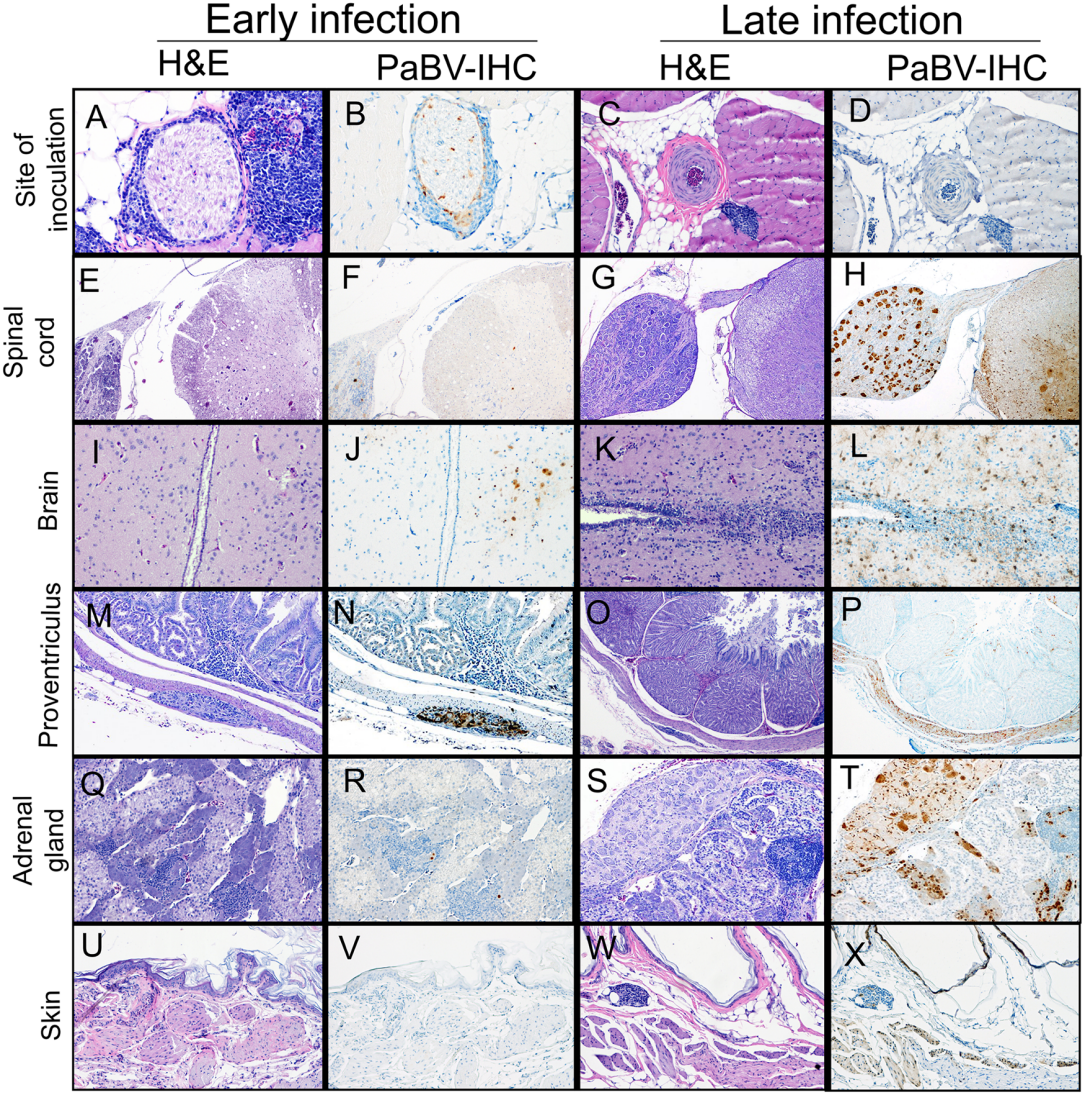
Sequence of histological findings and immunohistochemical labelling in tissues from infected cockatiels during early and late infection. The first tissue to present lymphoplasmacytic inflammation and positive labelling was the site of inoculation, which showed marked lymphoplasmacytic myositis (A) and positive immunolabeling in the nuclei of inflammatory cells as well as in Schwann cells of intercostal and pectoral nerves (B) in early infection (CK2, 5 dpi) and reduced inflammatory infiltrates (C) and no positive immunolabeling (D) at late infection (CK34, 114 dpi). PaBV then reached the spinal cord through adjacent ganglia and nerves, which provoked minimal myelitis (E) and infection of few neurons (F) in the early timepoints (CK9, 25 dpi) and severe inflammation (G) and widespread viral distribution (H) in late infection (CK26, 80 dpi). When PaBV reached the brain (CK10, 25 dpi), minimal inflammation (I) and positive immunolabeling of few neurons (J) was observed, whereas severe inflammation (K) accompanied by widespread immunolabeling (L) was observed in chronically infected cockatiels (CK30, 100 dpi). Only after invasion of the CNS, PaBV was identified at 30 dpi (CK19) in the GI tract (N), initially with mild inflammation (M) that progressed to moderate to severe ganglioneuritis (O) and diffuse immunolabeling in ganglia, smooth muscle and few scattered epithelial cells (P) in the late infection timepoints (CK28, 80 dpi). Adrenalitis was only observed in small areas of the adrenal medulla (Q) accompanied by scattered PaBV positive cells (R) in the early infection (CK14, 30 dpi), however, adrenalitis and ganglioneuritis (S) were severe in late infection (CK25, 60 dpi) and followed by wide spread immunoimmunolabeling (T) affecting the adrenal medulla and adjacent ganglia. Finally, tissues such as skin had no inflammation (U) or immunolabeling (V) in the early infection timepoints (CK7, 20 dpi) but presented multifocal positive immunolabeling pattern (X) in late infection (CK34, 114 dpi), accompanied by mild to moderate inflammation (W).

The distribution of PaBV-2 N-protein in different organs of infected cockatiels is shown in Table 1, and detailed information about each bird is included as supporting information (S1 Table). The site of inoculation was consistently positive throughout the timepoints, with lower signal with the progression of the infection and no signal at the last two timepoints. Lymphocytes and plasma cells presented positive intranuclear immunolabeling and macrophages presented intracytoplasmic labelling in these sites (Fig 4B). Nuclei of Schwann cells in the branches of the intercostal and pectoral nerves were also positive in some of the early timepoints (Fig 4B). Brachial plexus was the first site outside of the pectoral muscle to demonstrate intranuclear immunolabeling in Schwann cells starting at 20 dpi. Neurons of the dorsal root ganglia had a similar immunolabeling at this timepoint (Fig 4F). Neurons of the ventral horn in the thoracic segment of the spinal cord were the first cells in the CNS to present positive intranuclear and intracytoplasmic immunolabeling at 20 dpi. After 25 dpi, neurons and glial cells in the cervical and lumbar segments of the spinal cord as well as throughout the brain presented intranuclear and intracytoplasmic immunolabeling (Fig 4J). These sites were consistently positive until the end of the experiment, varying in severity and extension. Simultaneously at 30 dpi, neurons of the ingluvial and intestinal ganglia were the first immunolabeled cells in the gastrointestinal tract along with adrenal ganglia neurons and cells of the adrenal medulla (Fig 4R) were positive. Ganglia and nerves of the proventriculus (Fig 4N), ventriculus, and eyes as well as the vagus nerve were first positive at 35 dpi. Esophageal, epicardial, renal and cloacal ganglia were first positive at 40 dpi. At 60 dpi, scattered cells of islets of Langerhans of the pancreas were positive, which were intermittently positive until 114 dpi. Interestingly, at 80 dpi, nerves and the smooth muscle in the skin (Fig 4X) from multiple regions (behind the neck, wing and uropygial gland) and also the smooth muscle of crop, proventriculus, ventriculus, intestines, cloaca and tunica media of vessels from different tissues, including the spleen, started to have a diffuse immunolabeling that was intermittently positive until the last timepoint. Epithelial cells of the GI system also showed few scattered intranuclear immunolabeling at this timepoint. Additionally, nerves and smooth muscle of the feathered skin between the scapulae and the squamous epithelium and occasionally the feather shaft of feather follicles from the wing started to be positive at 100 dpi and 114 dpi, respectively. Samples from tongue, trachea, lungs, head sinuses, syrinx, thyroid, parathyroid, tibiotarsus, or thymus, were negative at all timepoints and no control cockatiels presented immunolabeling in any of the examined tissues.

### RT-PCR

The detection of PaBV-M gene RNA is shown in Table 1. All samples from the site of inoculation for the timepoints between 20 and 114 dpi were positive for PaBV RNA by PCR. The brachial plexus, spinal cord, brain, crop, proventriculus, ventriculus, small intestine, large intestine, adrenal gland and heart/great vessels of the heart RT-PCR results were positive and correlated to the IHC positive results. Cloacal swabs were first positive at 35 dpi and choanal swabs at 60 dpi, and both samples remained positive until 114 dpi. Although the feathered skin between the scapulae was negative by IHC until 95 dpi and the skin behind the neck and from the wing were negative until 80 dpi, these samples were positive by RT-PCR as early as 60 dpi. Samples from spleen were positive at 100 and 114 dpi. Samples from blood and liver were collected and tested by RT-PCR but were negative at all timepoints. Unfortunately, samples from the site of inoculation were not available at 5 and 10 dpi. All samples from control cockatiels were negative at all timepoints.

Real time PCR was one serial dilution more sensitive than conventional PCR when 10-fold dilutions were compared between the 2 techniques, therefore selected samples from cloacal and choanal swabs, brachial plexus, spinal cord and brain for early time points, and samples from blood and liver of all cockatiels were tested with this more sensitive technique in order to confirm the accuracy of conventional PCR results. For the early time points, samples of cloacal and choanal swabs, brachial plexus and spinal cord tested positive in additional cockatiels that were negative by conventional PCR. However, these cockatiels were within timepoints where other birds were already positive by conventional PCR. One exception was the choanal swab, which was positive in one cockatiel (CK22) at 40 dpi, while conventional PCR first detected it at 60 dpi. Viral RNA was only detected in samples of spleen from cockatiels that were positive by conventional PCR. All samples of liver and blood tested negative at all time points. A complete list of the qPCR results can be found as supporting information (S2 Table).

## Discussion

**Fig 5:**
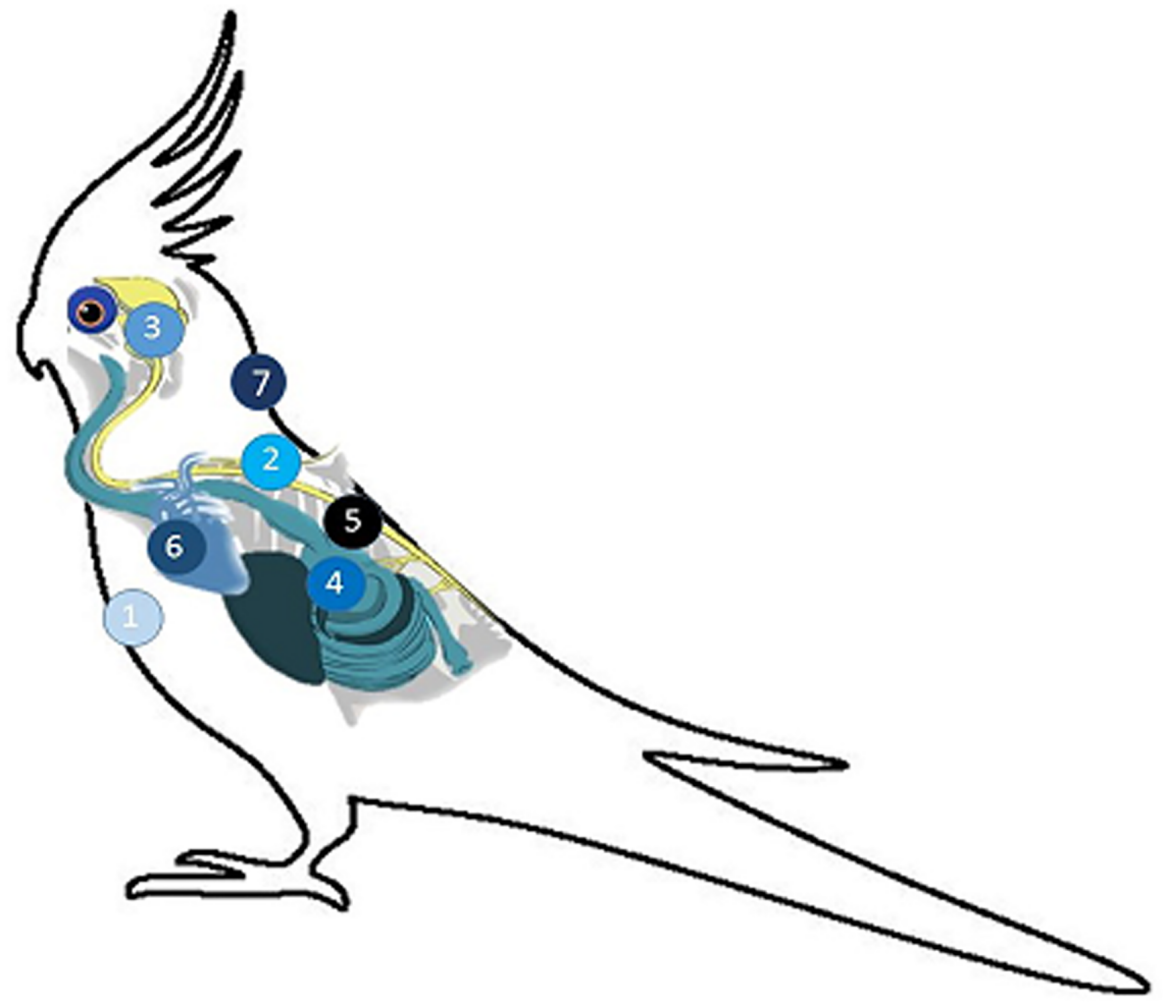
Chronologic infection pathway based on PaBV N-protein detection by IHC. Positive immunolabeling after intramuscular inoculation (1) was first observed at 20 dpi in the spinal cord (2), followed by brain (3) at 25 dpi. Ganglia in the GI tract (4) and adrenal gland (5) were first positive at 30 dpi. Positive immunolabeling in the epicardial ganglia (6) was first observed at 35 dpi. Extra-neural tissues such as skin (7) only had positive immunolabeling at late infection timepoints, as early as 80 dpi.

This is the first experimental study to demonstrate the viral spread and distribution of histological lesions in a time-based approach after intramuscular inoculation of PaBV-2 in cockatiels (Fig 5). PaBV-2 was first detected in the areas of inflammation in the site of inoculation in the pectoral muscle, reaching branches of the intercostal and pectoral nerves. Subsequently, PaBV-2 reached the brachial plexus, and centripetally extended through the dorsal root ganglia to the spinal cord, which was followed by the invasion of the brain. After successfully invading the CNS, PaBV-2 centrifugally spread to the ganglia in the gastrointestinal system, adrenal gland, heart and kidneys through the sympathetic and/or parasympathetic innervation. Finally, a shift in the specific tropism of the virus was observed in the late timepoints, and not only nerves and ganglia were affected but also the smooth muscle and/or scattered epithelial cells of tissues such as crop, intestines, proventriculus, kidneys, skin, and the tunica media of the vessels culminating in a widespread viral distribution.

Previous experimental studies with PaBV-2 and PaBV-4 have been able to successfully induce clinical disease, and the classical meningoencencephalomyelitis and ganglioneuritis [2, 8, 19, 20, 24, 26–28]. However, whether the virus spread to the CNS from the PNS or vice-versa was never clearly understood since in these studies the birds were only euthanized when they presented clinical disease or at the end of the experiment. Therefore, these investigations analyzed early infection aspects only by *intra vitam* parameters such as viral shedding and seroconversion, and important information about the viral pathway that was present in the early infection, and could have been detected by histopathology and immunohistochemistry, was consequently missed. Interestingly, we first detected viral RNA in cloacal swabs at 35 dpi and in choanal swabs at 60 dpi, which contrasts with some experimental studies in canaries and cockatiels where viral shedding was detected intermittently after the first week post-inoculation [28, 29]. However, important factors such as the species used in the experiment and mixed routes of experimental infection must be taken into consideration for explaining this earlier viral shedding. Additionally, other studies using intramuscular and subcutaneous route of infection in cockatiels were able to first detect viral RNA in swabs between 7 and 11 weeks post-inoculation, which corroborates with our results [27].

The natural route of infection of PaBVs is a nebulous aspect of PDD pathogenesis. Experimental studies were able to infect psittacine birds using single intramuscular, intravenous or intracerebral routes [2, 20, 30] or using different protocols of intramuscular, oral, oculonasal, and subcutaneous routes combined [9, 19, 24, 26]. Recently, two studies involving cockatiels inoculated by oculonasal or oral routes failed to establish persistent PaBV-2 or PaBV-4 infection or to induce PDD lesions in any of the birds during 6 months of experiment [31] suggesting that these routes are unlikely to be involved in natural PaBV-2 infection. Furthermore, in our experiment, the successful induction of PaBV-2 infection through intramuscular inoculation, and the widespread distribution of PaBV-2 in late infection timepoints indicate that direct transmission of PaBV-2 through trauma wounds or tegument injury might play a pivotal role in the pathogenesis. A novel bornavirus recently identified in variegated squirrels (Variegated squirrel bornavirus-1; VSBV-1) was linked to three cases of fatal encephalitis in humans who were breeders of this squirrel species [32]. Although the route of infection still remains unclear in these cases, family members reported skin injuries due to squirrel bites and scratches for 2 of the patients, which indicates that this route of infection might be involved in the VSBV-1 transmission. Interestingly, VSBV-1 RNA was observed in several tissues of the variegated squirrels that had contact with the breeders, including brain, heart and kidneys, and VSBV-1 RNA and antigen were identified in the neurons, glial cells and neuropil in the brains of the breeders. The hypothesis that VSBV-1 could be transmitted via skin injuries is further supported by a recent study where 11 out of 468 squirrels originated from private institutions, zoological gardens and roadkill were screened in Germany and Netherlands and tested positive for VSBV-1 RNA with the highest loads in the CNS, oral cavity and skin [33]. Furthermore, the presence of VSBV-1 in the oral cavity of infected variegated squirrels could be associated with transmission of this virus to humans in eventual biting accidents.

Neurotropic pathogens can invade the brain by different strategies, including retrograde axonal transport, hematogenous spread across the blood-brain barrier (BBB), direct infection of endothelial cells and via spread of infected leukocytes across the BBB into the parenchyma, also known as “trojan horse” mechanism [34]. In the early timepoints of our experiment, PaBV-2 was restricted to branches of pectoral muscle nerves, brachial plexus, neurons of the dorsal root ganglia and spinal cord, even before inflammation could be observed in the latter two tissues. Only after reaching the brain, PaBV-2 was observed spreading to extra-neural tissues. Our results suggest that PaBV-2 reaches the CNS through retrograde axonal transport in a similar way to another neurotropic negative sense single-stranded RNA virus such as Rabies virus (RABV). RABV is transmitted by the bite of infected animals, infects motor neurons at the neuromuscular junctions in the site of injury, and then spreads to the spinal cord by retrograde axonal transport, which eventually leads to infection of the brainstem and limbic system [35]. Once in the brain, RABV migrates throughout the CNS and then back to the periphery via anterograde axonal transport. Late in infection, the virus can be seen in the skin, salivary glands, heart, kidney and cornea [36]. Experimental studies have provided evidence that Borna disease virus 1 (BoDV-1) uses nerve endings in the nasal and pharyngeal mucosa to gain access to the olfactory bulb and consequently to the brain via retrograde axonal transport and can infect neurons, astrocytes, ependymal cells and oligodendrocytes of experimentally infected rodents [37]. In contrast to the scenario seen in PaBV reservoir hosts, mammalian Bornaviruses are strictly neurotropic and encephalitis is the most striking manifestation of Bornaviral infection in its dead-end hosts, including horses, sheep, cats, and rodents [36]. In contrast, in the bicolored shrew (*Crocidura leucodon*), the natural reservoir of BoDV-1, this Bornavirus was identified in several neural and extra-neural tissues, and continuously shed through secretions, excretions, and even skin scalping [38]. BoDV-1 infection in experimental and erroneous hosts is mainly restricted to the gray matter of the brain and spinal cord, and despite vision impairment has been mostly attributed to central blindness [37], degeneration of retinal neurons has also been reported [39]. Other neurotropic agents such as Poliovirus [40] or Scrapie prions [41] may also use retrograde axonal transport to spread to the CNS, however, in these cases, viremia and/or infection of the gastrointestinal associated lymphoid tissue (GALT) occur after oral ingestion of these agents, which precedes invasion of CNS.

The combination of immunohistochemistry and molecular methods used in our experiment was able to provide valuable information about viral antigen localization and its relationship with viral RNA. For instance, although samples from spleen were positive by PCR at 100 and 114 dpi, IHC revealed that only smooth muscle of the splenic vessels were positive, which corroborates with the late infection pattern seen in our study and agrees with the results of other studies that were able to demonstrate only low viral loads in this tissue [20, 27]. Additionally, we could not detect viral antigen nor viral RNA in any samples of liver, which also corroborates with other studies that demonstrate very low or no load of PaBV-2 in this tissue [27, 28].

Although a slow growing virus in cell culture, PaBV-2 detection can be observed *in vivo* as early as 20 dpi, using an intramuscular route of infection. Interestingly, despite the hallmark lesion of PaBV infections being the dilation of the proventriculus, our results demonstrated this virus affects the CNS at least 10 days before migrating to peripheral tissues like the GI system. In the late infection, viral tropism for neural tissue is not as strict as in the early infection timepoints, and PaBV-2 has broad tissue tropism affecting extra-neural tissues. Our findings reinforce the potential of samples for antemortem diagnostic methods such as crop immunohistochemistry and cloacal swabs PCR for detection of PaBV-2 in early infection timepoints, as early as 30 and 35 dpi, respectively. Additionally, skin samples and choanal swabs might be useful for detection of PaBV-2 by RT-PCR in chronically infected patients. On the other hand, we were not able to detect PaBV-2 RNA in the blood of any of the cockatiels in this experiment, which corroborates with other studies that concluded that blood samples are not reliable for PaBV-2 detection by RT-PCR [18, 26, 42] and viremia is unlikely to take place in the pathogenesis of PDD. Future research is necessary to understand key features of PaBV-2 infection and PDD pathogenesis such as natural route of infection, receptors in target cells, and immune response.

## Supporting information

S1 Fig Lack of immunolabeling in non-infected cockatiels. Ventricular ganglion (A), Epicardial ganglion (B), Spinal cord (C), Cerebellum (D), Adrenal gland (E) and Cerebrum (F).
(JPG)

S1 Table Individual results of histopathology, immunohistochemistry and conventional PCR for all cockatiels. (XLSX)

S2 Table Real-time PCR results for selected samples of infected cockatiels. (XLSX)
