# Supplementary figures and images for: From nerves to brain to gastrointestinal tract: A time-based study of parrot bornavirus 2 (PaBV-2) pathogenesis in cockatiels (Nymphicus hollandicus)
Source: PLoS One. 2017 Nov 9;12(11):e0187797. doi: 10.1371/journal.pone.0187797 (PMC5679548; doi:10.1371/journal.pone.0187797)

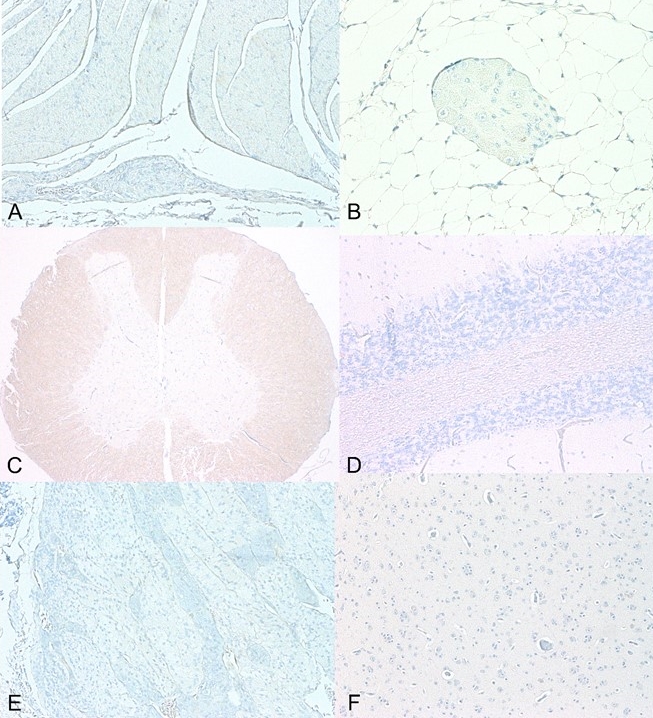

Supplement: S1 Fig — Ventricular ganglion (A), Epicardial ganglion (B), Spinal cord (C), Cerebellum (D), Adrenal gland (E) and Cerebrum (F). (JPG) [file pone.0187797.s001.jpg]
